# Effect of Thermal Degradation of FKM on Three-Body Abrasion under Dry Sliding: Severe Damage Led by the Particle Detention

**DOI:** 10.3390/ma14143820

**Published:** 2021-07-08

**Authors:** Kun Qin, Qin Zhou, Kai Zhang, Minghao Lv

**Affiliations:** Key Laboratory on Deep Geo-Drilling Technology of the Ministry of Natural Resources, School of Engineering and Technology, China University of Geosciences, Beijing 100083, China; 3002180019@cugb.edu.cn (K.Q.); 2102190019@cugb.edu.cn (M.L.)

**Keywords:** three-body abrasion, thermal degradation, particle motion, sealing component in drilling, fluororubber (FKM)

## Abstract

Both the high temperature and particle environment at the downhole greatly aggravate the abrasive wear and shorten the service life of the fluororubber (FKM) seal seriously in drilling engineering. At present, there is less awareness of the tribological behavior of seals in such complex working conditions. In this work, the abrasive wear performance of the thermally degraded FKM seal was tested in the form of simulating the intrusion of abrasive particles into the interface. Results show that the wear of both rubber seals and metal counterparts is exacerbated. Through the analysis of the wear scar morphology and friction coefficient, it is revealed that more abrasive caves scatter on the surface due to the mechanical degradation of the FKM. These abrasion caves reduce the tendency of particles to escape from the caves and prolong the abrasive action. Furthermore, the abrasion cave alters the particle motion from sliding to rolling, which leads to more caves generated on the surface of the hard tribo-pair. These results enhance the understanding of the abrasive wear for FKM seals and hopefully contribute to the promotion of seals used in hot abrasive particle environments.

## 1. Introduction

In drilling engineering, rubber seals are widely used to prevent drill cuttings from intruding into the seal chamber of the drilling tools, such as the roller bit, rotation guide tools, devices of measurement while drilling, etc. [1,2]. However, the working performance of the downhole seal is not satisfactory. It has been found that the service life of the rubber seal used under drilling conditions decreases significantly. The rapid failure of rubber seals is further exacerbated when it is used in the deep well, ultra-deep well drilling, and geothermal well drilling [3,4,5,6]. What these working conditions have in common, except the abrasive condition, is the high ambient temperature [7]. For example, the well temperature could achieve 150 °C when its depth reaches 5000 m (geothermal gradient: 25–30 °C/km). In recent years, with the gradual exhaustion of shallow resources and increasing needs for geothermal resources, more and more sealing components in drilling have to work in an abrasive particle environment while affected by the hot temperature. Therefore, to effectively improve seal durability, it is important to understand the effect of high temperature on the tribology behaviors of seals in abrasive environments.

Working in an abrasive condition, the abrasive wear led by the particles invading the sealing interface is considered to be the main cause of the accelerated wear failure of the seal. During the abrasive wear, the particle motion form is believed to play a critical role in affecting the friction and wear of the rubber [8,9,10,11]. When the abrasive particles are embedded on the sealing pair (rubber or steel), the fixed particles play the role of a cutting tool leading to two-body abrasion of the counter surface. In this condition, the particle motion is mainly sliding. When the particles slide against the rubber sealing face, the typical wear properties change with the particle size, which is known as the ‘Particle size effect’ [12,13]. In contrast, when the state of particles in the friction interface is free, the particle motion includes sliding and rolling at the same time. The movement of the particle can be confirmed by considering the normal force and the lateral force of the particles and their movement. Regardless of the different movements of the particles, the wear loss (material removal) of the seal is related to the shearing and stretching effects of the abrasive particles on the rubber. The mechanical properties are thus greatly affecting the tribology behaviors of rubber seals.

However, under the effect of high temperature, it was observed that the mechanical properties of the rubber material altered. As the polymer, the molecular link of the rubber is easy to break due to the hot environment, which increases the cross-linking density [14,15,16]. Moreover, some of the broken molecular links form a new fragile molecular chain (compared to the chain before the break) through cross-linking. It is reported that these chains can only withstand lower stress [16]. The stability of the structure of the newly cross-linked polymer network is also decreased. Therefore, the mechanical properties of the rubber elastomer gradually fade, which further leads to a decrease in the shear and stretch resistance [17]. This is the degradation of the rubber led by the high temperature. Hence, it could be expected that the wear loss of the degraded rubber should increase; however, it is surprising that the wear rate of the two-body abrasion of the rubber is decreased. In an unpublished study, it has been found that this is due to the fact that the degradation of rubber changes the friction and wear mechanism in the stable state from a severe surface tear to a slight micro-cut. At present, although the deterioration of the three-body abrasive wear performance of rubber components under thermal degradation has been noticed, its mechanism is still not clearly understood [18]. This situation greatly limits the development of new products with better performance.

As is well known, the thermal degradation of rubber is not the only factor that affects the sealing performance, which also includes, for example, drilling mud, lubricating oil, various abrasive particles, and other possible interference factors. However, the significant deterioration of the service life of rubber seals under high temperatures indicates the criticality of understanding the effect of thermal degradation of the rubber seals on the tribology behaviors of three-body abrasion. Therefore, the aim of this study hopes to lay a foundation for the study of the tribological behavior of seals operating under drilling conditions or other thermal abrasion conditions. Thus, in this study, the three-body abrasive wear test results of thermally degraded rubber seals and untreated seals were compared to investigate the effect of the thermal degradation of rubber on the tribological behaviors. The results gained from this work could provide experimental support for further study on the interactions of rubber seals and abrasive particles in more complex environments.

## 2. Materials and Methods

### 2.1. Materials

The tribo-pair is composed of a rubber seal and a metal disc. The test seal is an O-ring seal, and its material is FKM, which was made by blending vinylidene fluoride (80 wt %) and hexafluoropropylene (20 wt %). Micro silica (SiO_2_) particles were used to reinforce the rubber matrix. Yanglei Rubber & Plastic Co., Ltd. (Shanghai, China) provided seals for this research. The Poisson’s ratio of the FKM is 0.48, the modulus of elasticity is 7.8 MPa, the hardness is 80 (Shore A), and the density is 2.2 g/cm^3^. The modulus of elasticity was measured and calculated according to the standard test method of HG-T 3321-2012 and provided by the sample manufacturer. The surface roughness of the rubber seal is 1 μm. The metal disc is made of standard steel (ASTM Standard Steel No. 304), which was purchased from the Stainless Steel Trading Company of Lurui (Tianjin, China). The surface roughness of the steel disc is 0.1 μm. The dimensions of the metal disc and the FKM seal ring are displayed in Table 1.

**Figure 1:**
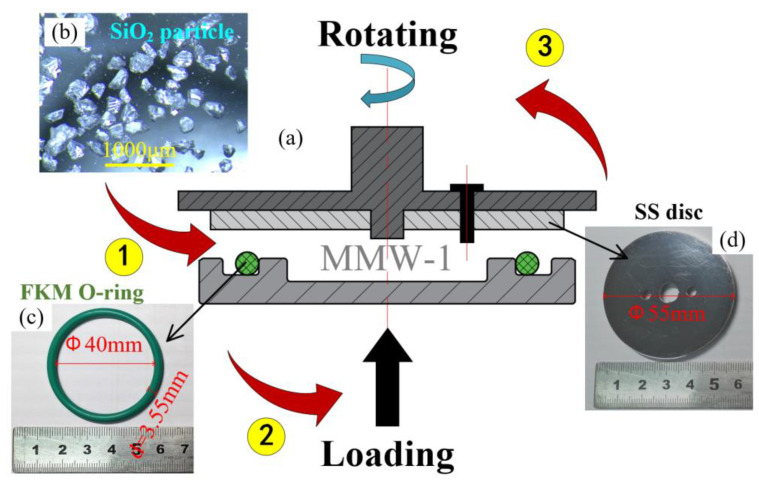
Schematic diagrams of (**a**) MMW-1 wear test rig (not to scale), and the inserts are (**b**) the optical micrograph of abrasive particles and (**c**) the pictures of the FKM seal and (**d**) metal disc, respectively.

The abrasive particles used in the three-body abrasion tests were supplied by the Fucai Mineral Products Material Co., Ltd. (Lianyungang, China). The composition of the hard particles is mainly SiO_2_, which is also the main component of rock. The SiO_2_ particles have a Mohs hardness of 7. After passing through the 70 mesh sieve, the particle size is 212 ± 10 μm. Figure 1a shows the optical microscope image of abrasive particles and the optical image of the FKM seal and metal disc.

### 2.2. Thermal Treatment

In drilling engineering, it takes about 7 h for a new seal to reach the bottom of the well and start working. In this process, the temperature experienced by the sealing O-ring gradually rises and eventually reaches 150 °C. However, due to the different geothermal gradients in different regions, the time for the sealing ring to be exposed to 150 °C is also different. In order to simulate the harshest environment, the sealing ring was exposed to a high temperature of 150 °C throughout the entire process. For indoor experiments, the FKM seal was heated in a chamber electric furnace (Beijing Mingchuang Oven Co., Ltd., Beijing, China) at 150 °C. Due to the short service time in the hot environment, the time of thermal treatment was set to seven hours. Then, to ensure the stability of the material properties, the treated seals were exposed to the room temperature for 24 h. The same heat treatment was also performed on standard dumbbell-shaped and crescent-shaped samples. After the thermal treatment, these two types of standard samples were used to measure the tensile strength, elongation at break, and tear strength of treated FKM and untreated FKM according to ISO 37-1994 and ASTM D624-00 (2012).

### 2.3. Wear Tests Rig and Procedures

To reveal the effect of the thermal treatment on the tribology behaviors, the test was divided into two groups: the treated group and the untreated group. The wear tests of treated specimens (TS) and untreated specimens (UTS) were performed under dry sliding conditions using the MMW-1 multi-function tester (Figure 1a), which is produced by Jinan OuTuo Test Equipment Co., Ltd. (Jinan, China). By real-time recording of the torque and normal force of the friction pair during the friction process, the friction coefficient can be automatically calculated according to the standard of ASTM D3702-94.

During the drilling process, when the rock debris intrudes into the sealing interface, the abrasive particles do not leave the contact area immediately but re-circulate. The three-body abrasion of the sealing interface is thus considered a closed system [19]. This experimental method has been introduced in our previous research [20]. As shown in Figure 1b, the abrasive particles were first sprinkled on the surface of FKM seals evenly to simulate the intrusion of particles into the sealing interface.

Then, the normal force of 180 N was applied to the rubber specimens to make it contact with the metal disc, which resulted in a compression ratio of 8% of the rubber seal. The compression ratio (*r*) of the O-ring can be calculated as the following equation:

*r* = *d* − *h/d* (1)

where *d* is the section diameter of the O-ring, which is the thickness of the O-ring before loading; *h* is the thickness of the O-ring after loading. Finally, the disc was rotated by an AC motor at a linear velocity of 0.42 m/s. The test mode in the current research was to sprinkle the particles on the rubber indicator in advance instead of sending them into the interface in real time. To avoid the wear characteristics of the three-body abrasion from being covered up, the test time was set to 200 s. After the experiments, the scanning electron microscope (ZEISS Gemini SEM, Jena, Germany) and the 3D measuring laser microscope (Olympus OLS4000, Tokyo, Japan) were used to examine the worn surface morphologies of the rubber and metal specimens. Since rubber is not conductive, the worn surface was first subjected to ion sputtering treatment before the SEM test, but this treatment did not affect the test results.

**Figure 2:**
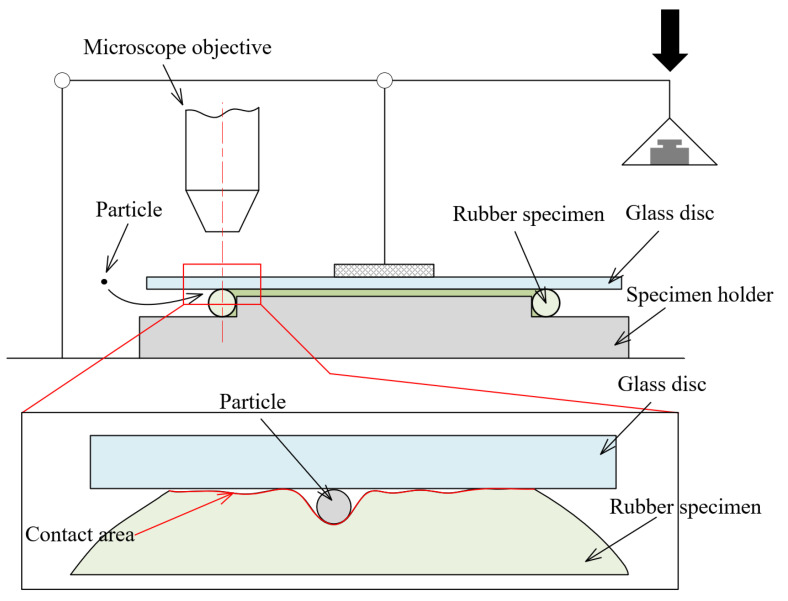
Schematic diagrams of the in situ observation device (not to scale).

Considering the difference in contact widths caused by possible changes in hardness, an in situ device was designed to test and measure the contact area between the FKM O-ring and the hard counterpart. As shown in Figure 2, a glass disc was used to replace the metal disc so that the contact area could be observed, and the normal load was applied to the glass disc by the lever. A microscope (Olympus SZX7, Tokyo, Japan) was used to observe the soft–hard contact area in situ.

## 3. Results and Discussion

### 3.1. Degradation Effect on Mechanical Properties

**Figure 3:**
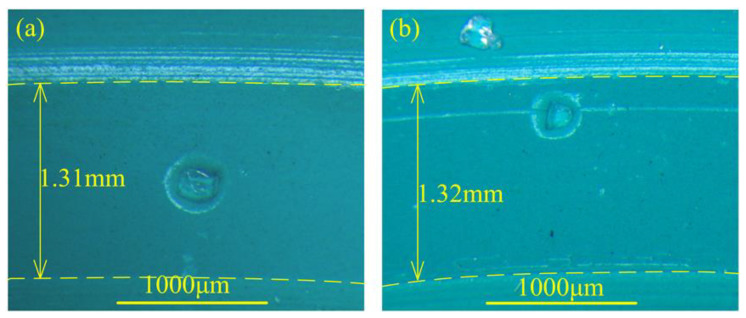
The optical micrograph of in situ observation of the contact area of (**a**) untreated seal and (**b**) thermally treated seal.

During the abrasion of a soft–hard contact pair, the surface tear and stretch are always concerned. Moreover, the tribology performance is believed to depend on the mechanical properties. Therefore, after the thermal treatment of the FKM specimens, the values of elongation at break, tensile strength, tear strength, and shore hardness were measured, and the average values from at least five repeated operations were recorded in Table 2. The results show that although thermal treatment significantly reduces the tear strength, tensile strength, and elongation at break, the hardness of the rubber does not change significantly but only slightly increases. For a soft–hard contact pair, the hardness change of the soft counterpart may influence the contact condition. The contact areas of the two kinds of specimens are shown in Figure 3. The sizes (widths) of the contact areas are almost identical, which shows that the effect of the thermal treatment on the contact area is not significant [21].

In past research, the change in mechanical properties caused by high-temperature treatment was considered to be caused by the thermal degradation of the rubber [14,15,16]. It is reported that under the effect of high temperatures, the FKM undergoes a cross-linking reaction. In this process, the original molecular bonds of the FKM are broken, and new but fragile molecular bonds are formed at the same time. The force that the degraded molecular bond can withstand is reduced, which leads the treated FKM to be easily broken or torn. In addition, some studies have found that the cross-linking reaction caused an increase in the cross-link density, which was further believed to increase the hardness of the FKM [16].

### 3.2. Coefficient of Friction

**Figure 4:**
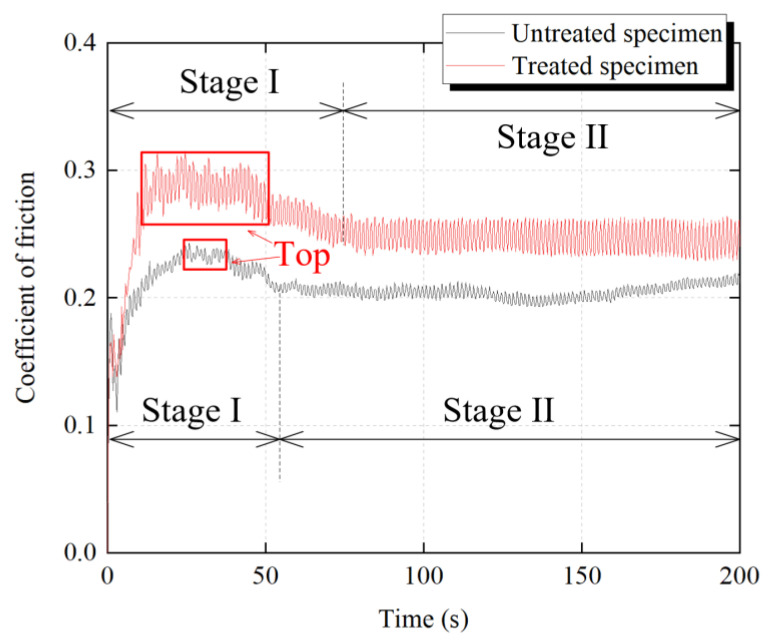
Coefficients of friction between the metal disc and untreated specimens and treated specimens.

After at least three tests, the identical evolution law of the coefficient of friction (COF) as a function of time was observed, and the representative results of TS and UTS are shown in Figure 4. The same trend can be found in both curves, i.e., the COF was achieved at the top then decreased to stabilization gradually. For UTS, the highest value of COF was about 0.24, and the steady value was about 0.2. In contrast, for TS, the highest value of COF was about 0.29, and the steady value was about 0.25. When the COF reached the top, the friction coefficient of the TS obviously continued for a longer period compared with the UTS, as shown in Figure 4 Through the whole test, the friction coefficient of the treated group was in an unstable state (stage-I) for longer.

### 3.3. Worn Surface

**Figure 5:**
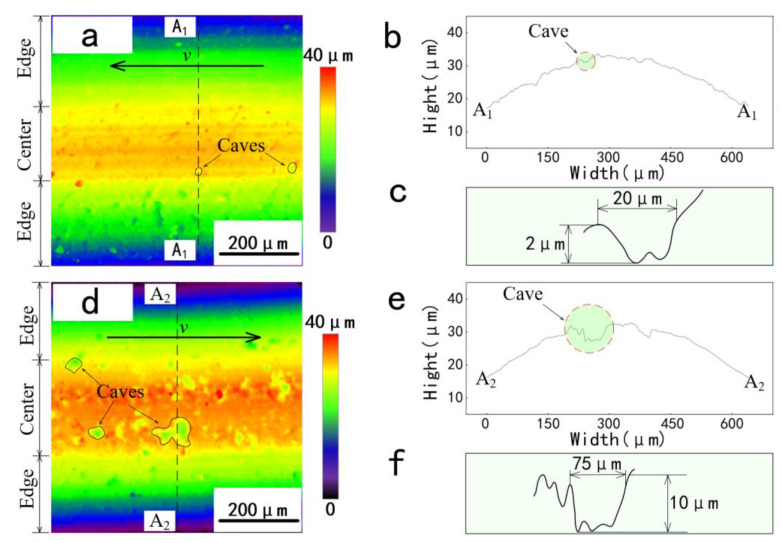
Top views of the three-dimensional topography of (**a**) untreated specimens and (**d**) treated specimens after wear tests and their two-dimensional profiles of (**b**,**e**) worn scars (marked with dashed lines A_1_-A_1_ and A_2_-A_2_) and (**c**,**f**) caves.

To understand the differences in COF, the wear surface of the tribo-pair was investigated. Figure 5a,d shows the top views of the three-dimensional topography of the worn surface. On the wear surface, higher areas were marked with warm colors, while lower areas were marked with cool colors, and areas with similar heights were marked with similar colors. Significantly, the height of the upper-end face of the worn surface was relatively uniform (marked in red or orange). The area with a warm color can be defined as the center area, and the area with a cool color can be defined as the edge area. Considering the section of an O-ring specimen is a circle (parabolic shape), the flatland of the center area reflects that the surface removal led by the three-body abrasion in this area was more serious than in the edge area. This is because for the contact pair of the O-ring seal and the metal disc, the highest contact pressure is located in the center, and the contact pressure along the sealing edge is lower. Under the high contact pressure, more wear volume was generated in the center area. Comparing the width of the center area of two kinds of specimens, a wider center area of TS may indicate that the wear of the thermally treated FKM O-ring seal is more serious.

In the center area, spots with cool colors can be found. The spot in Figure 5a is significantly smaller and less than that in Figure 5d. To confirm the depth of the spot, the two-dimensional profiles of the worn surface were measured at the positions marked by the dashed lines A_1_-A_1_ and A_2_-A_2_. As shown in Figure 5b,c,e,f, the depth of the spot on the untreated specimen is shallower than that on the treated specimen. After several measurements, it was found that the depth of the spots on the treated specimens was in the range of 2–5 μm, while the depth of the untreated specimens was in the range of 5–12 μm.

**Figure 6:**
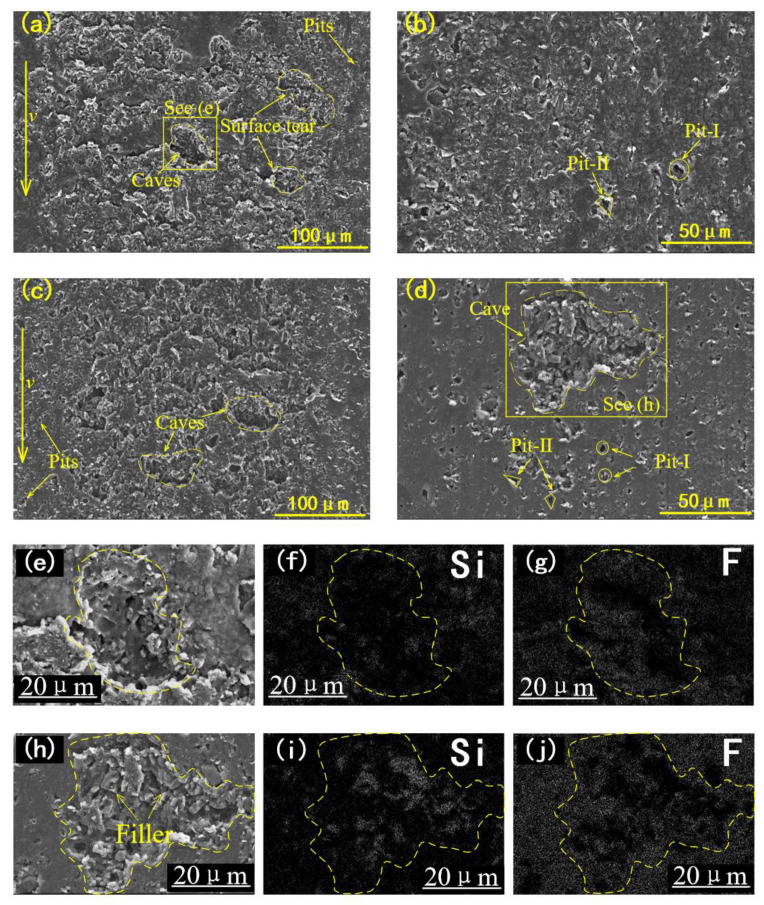
SEM micrograph of the worn surface of untreated specimens (**a**) in the center area and (**b**) in the edge area; the wear surface of treated specimens (**c**) in center area and (**d**) in edge area; (**e**) the cave in the center area of untreated specimens and its element mapping of (**f**) silicon and (**g**) fluorine; (**h**) the cave in the edge area of treated specimens and its element mapping of (**i**) silicon and (**j**) fluorine.

To further confirm the wear characteristics, the wear scars of the rubber O-rings were scanned by the scanning electron microscope. The SEM micrographs of the worn surface of two kinds of specimens in the center area and edge area are shown in Figure 6a–d.

At the center of the untreated specimen, two kinds of wear morphologies can be observed (Figure 6a). One is the parallel waves that are perpendicular to the sliding direction, which is known as the Schallamach-type wave (STW) [22,23,24]. Another wear morphology is the micro-scar with an ellipse-shaped contour (marked with the dotted lines). Some of the micro-scars are relatively deeper, which can be defined as the cave. The same wear morphologies are also found on the wear scar of the thermally treated specimens. The difference is that the cave almost dominates the wear scar of the TS, while the wave rarely appears. According to the top view of the 3D topography (Figure 5), the number and the size of the caves of TS are both more remarkable than that of UTS. In addition, the two-dimensional surface profiles of the wear surface indicated that the cave on the surface of the TS was deeper than that of the UTS, as shown in Figure 5b,e. The depth of the cave on the untreated specimens is about 2–5 μm (Figure 5c), while on the treated specimens it is about 5–12 μm (Figure 5f).

At the edge of the contact area, the wear morphology of the micro pit is found, whose size is about 1~5μm. The outline of some pits was rounded (pit-I), while some pits’ outlines were sharp (pit-II). Significantly, more pits were found on the surface of treated specimens. The cave is also observed at the edge area of the TS, but no significant cave is found in the same area of the UTS. It was noticed that the morphology of the bottom of the caves at the center area and the edge area was not the same. As shown in Figure 6e, pits with a round outline are found at the bottom of the cave observed in the center area. While in the edge area, small particles are observed at the bottom of the cave beside the pit (Figure 6h). To verify the identity of the particles, the energy dispersive spectrometer was used to reveal the identity of small particles. The enriched Si element indicates that the small particles are SiO_2_ fillers (Figure 6e). Therefore, the micro pit found in the bottom of the cave at the center area may be led by the shedding of fillers. Similarly, pit-I may also be caused by this reason. Moreover, pit-II is caused by the penetration of the rubber surface. More pit-II observed on the surface of the treated specimens indicated that the treated specimen is easier to penetrate.

Overall, the wear scar at the center contact area is obviously more rough and jagged than the worn surface at the edge of the contact area. The wear characteristic that appeared in the center area and the edge area exhibited that the wear mode of the rubber specimens depends on the contact force applied to the particles. For example, as the contact force increases, the wear characteristics of the rubber surface deteriorate, gradually from surface penetration to surface tearing to surface flake peeling. This reflected that the damage degree at the center area is more serious than at the edge area, which agrees with the results shown in Figure 5.

**Figure 7:**
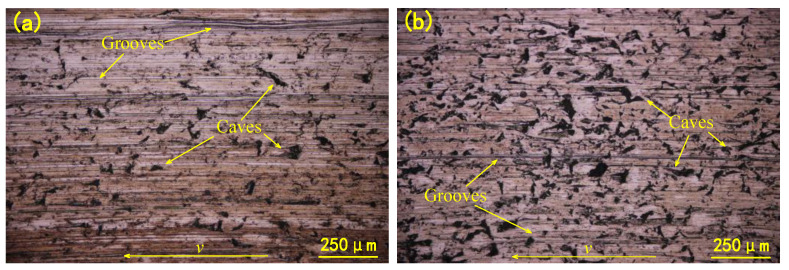
Wear surfaces of metal disc of (**a**) untreated specimens and (**b**) treated specimens.

Abrasive wear also caused the wear of the metal counterpart. As shown in Figure 7, the wear morphology of the stainless steel discs of the two groups is mainly grooves and caves. The grooves are parallel to the sliding direction, and the outline of the cave is sharp. The size of the cave on the SS disc was about 10–80 μm.

In three-body abrasion, the difference in the wear morphology of the hard tribo-pair is often related to the motion form of the abrasive particles. For example, it is believed that when the particles are embedded in the rubber surface, the particles are stationary relative to the rubber and scratch to form a furrow on the hard friction part. This is known as the grinding wheel effect [25]. In contrast, when free particles are rolling in the friction interface, the damage mode of the metal changes to the cave. These two particle movements, rolling and sliding, are dependent on the contact state of the abrasive particle. Compared with the wear track of the untreated group, significantly more caves were observed in the thermally treated group. This indicated that the particles tended to roll more in the frictional interface after the thermal treatment.

However, the wear characteristics that appeared on the rubber surface indicate that the motion of the particles is sliding, the same as the untreated specimens, which is inconsistent with the movement of the particles indicated by the wear morphologies of the metal surface. To understand the thermal treatment effect on the tribology behaviors better, it is necessary to analyze the particle movement.

### 3.4. Particle Motion

It is reported that the different movements of the particle depend on the force state of the particle in the friction interface. In the sliding interface, a particle is mainly subject to contact pressure and friction applied by the tribo-pairs.

**Figure 8:**
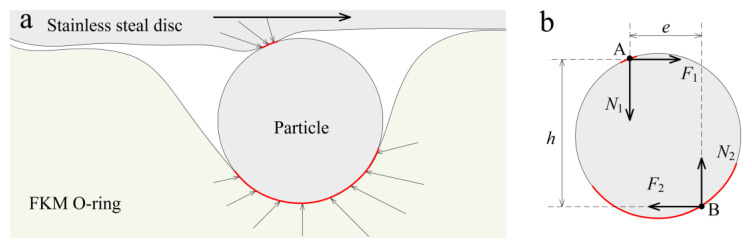
Schematic diagram of (**a**) forces on the particles in the three-body contact of the sealing interface, and (**b**) the sum of forces on an abrasive particle.

As illustrated in Figure 8b, points A and B are assumed to be the central contact points of distributed forces on the particle by the metal surface and rubber surface [9,10,11,12]. The total forces acting on the particles by the stainless steel disc are the frictional force (*F*_1_) and the normal force (*N*_1_) at point A, and the total forces acting on the particles by the FKM O-ring are the frictional force (*F*_2_) and the normal force (*N*_2_) at point B. When the particle is in equilibrium, it can be gained that

*F*_1_ = *F*_2_ = *F* (2)

*N*_1_ = *N*_2_ = *N* (3)

The torque *F *×* h* can be defined as the driving torque, which tends to make the particle roll, while the torque *N *×* e* is the resistant torque, which tends to resist rolling. In this case, when the is particle rolling in the friction interface, an expression is obtained as

*F* × *h* > *N* × *e* (4)

where the dimensions of *h* and *e* are the moment of *F* and *N*.

In contrast, when the particle is sliding in the frictional interface, the equation is

*F* × *h* ≤ *N* × *e* (5)

The normal force *N* is related to the deformation depth and the hardness of the friction pair. As shown in the in situ observation of the contact interface, the deformation depth, whether before or after aging, led by the particle did not significantly change. In contrast, the hardness of the FKM increased after the thermal treatment, although it was slight. The normal force *N* thus increased. Therefore, in the initial friction state, the particle motion is sliding.

**Figure 9:**
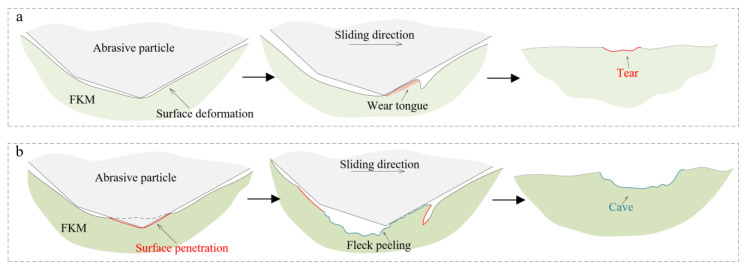
Wear mechanisms of (**a**) surface tear and (**b**) abrasion cave.

In this state, the wear mechanism of the particle sliding in the friction interface is illustrated in Figure 9. In the three-body contact, the abrasion makes the untreated rubber surface deformed. Then, under the sliding of the particle, the rubber surface is stretched and torn by the particle (Figure 9a). In this process, the wear loss mainly occurs at the root and the tip of the wear tongue. After the thermal treatment, the degradation of the mechanical properties results in the rubber surface being easier to penetrate. More minor surface defects, such as micro pits, were observed on the wear trace (Figure 6d). Then, with the sliding of the particle, the wear mechanism changes to fleck peeling (Figure 9b) [17].

**Figure 10:**
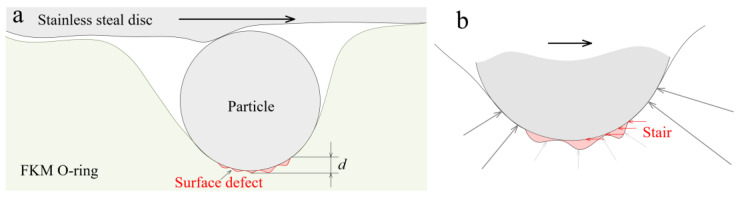
Schematic diagram of (**a**) the three-body contact with the surface defect, and (**b**) the forces on an abrasive particle contact with the surface defect.

After intruding into the sealing interface, the particles act as the third body and participate in the friction between the rubber seal and the hard counterpart. During the three-body abrasion, the particles do not leave the friction interface immediately but after a period of time. This means that the particles circulate at the sealing interface instead of once through. Therefore, a sealing interface belongs to the “closed group” of the three-body abrasion. The appearance of the defects, such as the cave and surface tear, changes the contact state of the particles (Figure 10a) during the re-circulation of the particles.

When another particle slides into surface defects, the contact force applied on the particle decreases because the compression amount of the rubber becomes smaller. The edge of the defect acts as, to some extent, a stair that prevents particles from leaving the defect (Figure 10b), which increases the lateral force. With the increase in the depth of the defect, it becomes more difficult for particles to slide. Therefore, the particles tend to strand in the defect during the re-circulation of the particles. This phenomenon is known as the collective effect of defects [26]. According to the 3D view of the wear morphologies (Figure 5), the size and depth of the caves are more remarkable, which increases the force that overcomes the resistance to sliding. Therefore, particles may be more likely to be collected in the cave.

During the retention of the particles, due to the cave reducing the normal force *F* and lateral force *N* applied on the particles, the motion of the particles that contact with the cave is more likely to be rolling in the interface rather than sliding Equation (4). The rolling particles scrape the cave, which may lead to the fillers remaining at the bottom of the cave falling off. It is worth noting that the size of the caves on the surface of the treated group is not significantly increased, only the number of caves is increased. This may indicate that when the particles stranded in the cave appeared on the rubber surface and rolled, the effect on the steel surface is to create more caves rather than to broaden the existing caves.

**Figure 11:**
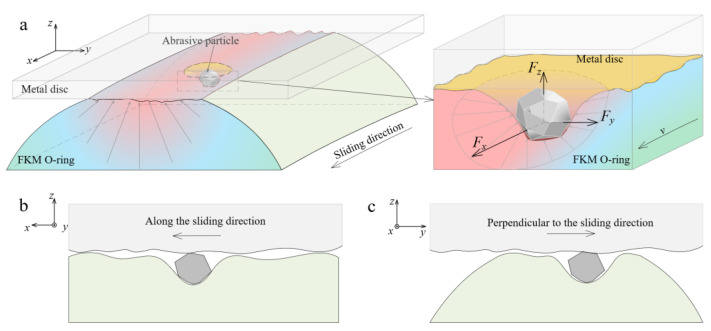
Schematic of (**a**) three-body abrasion of FKM O-ring, and the particle movement (**b**) along the sliding direction and (**c**) perpendicular to the sliding direction.

However, rubber fillers observed at the bottom of the caves were found in the edge area (Figure 6h). This indicated that the cave at the edge of the contact area seems to not have stranded the particles and, thus, was not scraped during the re-circulation of the particles. To clearly understand the trajectory of the particle, a simplified model of the three-body abrasion is illustrated in Figure 11.

Due to the cross-section shape of the O-ring being round, the contact stress gradually decreases from the center to the edge of the contact area (Figure 11a). When the particle rubs in the contact area, the resultant force applied on the particle that is perpendicular to the sliding (*F*_y_) always points to the direction that is away from the contact interface. With the effect of the *F*_y_, the trajectory of the particles means they gradually escape from the contact interface. Therefore, the two directions of the particle movement need to be considered: (i) movement parallel to the sliding direction (Figure 11b) and (ii) movement vertical to the sliding direction (Figure 11c).

The collecting effect of the cave on the particle delays the escape of the particles from the friction interface. Due to there being more caves generated on the surface of untreated specimens, more abrasive particles may strand in the tribological interface. This results in the friction interface being in a three-body abrasion. Hence, the frictional coefficient of a treated test group is in stage-I longer (Figure 4) compared to the untreated group.

In the edge area, as the normal contact force applied on particles (*F*_z_) is decreased, the restriction of the particles on the contact interface is reduced. It becomes easier for particles to escape the contact area. Therefore, the particles in the edge area tend to leave the frictional interface rather than re-circulate. Consequently, there are only a few pits in the edge area that develop into caves (Figure 6b).

### 3.5. Thermal Effect on Tribology Behaviors

The difference in tribological behaviors between thermally treated specimens and untreated specimens is mainly caused by the difference degree of surface damage led by the degradation of mechanical properties.

Firstly, reduction of the tear stress and tensile stress leads to aggravated damage to the friction surface, especially minor surface defects, such as pits. Due to the more particles that tend to penetrate the treated FKM surface, it should take more lateral force to achieve sliding. Therefore, the top value of COF of TS is larger than that of UTS.

Then, during the re-circulation of particles, the pit gradually evolved into serious surface damage, such as fleck peeling, which formed the cave. The appearance of the cave changes the movement of the particles, because when the particle contacts with the cave, the normal force is decreased while the lateral force is increased. The cave depth of the treated specimens is deeper than that of the untreated specimens, which leads the particles to be more inclined to roll in the friction interface of the treated group. Therefore, the caves led by the rolling of the particle are found more on the metal disc of the treated group compared with that of the untreated group. During the friction process, the particles gradually escape from the contact interface. However, the cave in the contact area could collect the particles, which delays the progress of the particle escape. That is to say, the particles were trapped in the contact area. Thus, the particles stay at the friction interface of TS longer than that of UTS. That is, the stage-I of COF of TS is longer than that of UTS.

Finally, a severely worn rubber surface and metal surface results in the friction coefficient of the stage-II of TS to be higher than that of UTS (Figure 4).

Generally speaking, the thermal treatment makes the FKM O-ring seal wear more serious, and worse surface wear leads to the particles stagnation in the tribo-interface and changes the particle movement from the sliding to the rolling. This further aggravates the surface wear of both rubber O-ring seals and metal counterparts. Of course, these results are not enough to fully explain the complex wear mechanism in the abrasive wear process, more work needs to be carried out to deepen the understanding.

## 4. Conclusions

This study investigated the effect of thermal treatment on the three-body abrasive wear of FKM seals and analyzed the movement of particles in the frictional interface. The following conclusions may be drawn from the results of this work:

Under the influence of high temperature, the mechanical properties of the fluororubber reduce, which results in more surface defects generated on the rubber surface. During the re-circulation of the abrasive particles, the minor defect gradually deteriorates, evolving from pits to surface peeling and finally forming caves. The abrasion cave reduces the tendency of particles to escape from the friction interface. More caves generated on the thermally treated specimens result in more particles detained in the contact interface, which prolongs the action of the abrasive particles. When the particle is collected in the cave, the motion of the particle is more inclined to roll in the frictional interface, which aggravates the surface damage of the metal counterpart.

In general, the thermal degradation of the FKM not only aggravates the wear of the O-ring seal itself but also aggravates the damage degree of its hard sealing counterpart. This discovery broadens the insight of sealing technology and should be taken into account in the development of a better alternative seal material used in a heat abrasive particle environment.

## Figures and Tables

**Table 1:** Dimensions of FKM rubber seals and stainless steel disc.

	Untreated FKM O-Ring	Stainless Steel Disc
Roughness (Ra)	1 μm	0.1 μm
Inner diameter	40 mm	-
Outer diameter	47.1 mm	55 mm
Thickness	-	7 mm

**Table 2:** The mechanical properties of the treated FKM specimens.

	Untreated Specimens	Treated Specimens
Elongation at break	410 ± 20%	360 ± 40%
Tensile strength	10.8 ± 0.4 MPa	9.2 ± 0.5 Mpa
Tear strength	17 ± 0.2 N/mm	15.4 ± 0.2 N/mm
Shore hardness	80.1 ± 1.1	82.3 ± 1.7

## Data Availability

This study did not report any data.
